# Cleft lip: The historical perspective

**DOI:** 10.4103/0970-0358.57180

**Published:** 2009-10

**Authors:** S. Bhattacharya, V. Khanna, R. Kohli

**Affiliations:** Department of Plastic Reconstructive and Aesthetic Surgery, Sahara India Medical Institute, Lucknow, India

**Keywords:** Cleft lip surgery, History of cleft lip, History of premaxilla management

## Abstract

The earliest documented history of cleft lip is based on a combination of religion, superstition, invention and charlatanism. While Greeks ignored their existence, Spartans and Romans would kill these children as they were considered to harbour evil spirits. When saner senses prevailed Fabricius ab Aquapendente (1537–1619) was the first to suggest the embryological basis of these clefts. The knowledge of cleft lip and the surgical correction received a big boost during the period between the Renaissance and the 19th century with the publication of Pierre Franco's Petit Traité and Traité des Hernies in which he described the condition as “lievré fendu de nativité” (cleft lip present from birth). The first documented Cleft lip surgery is from China in 390 BC in an 18 year old would be soldier, Wey Young-Chi. Albucasis of Arabia and his fellow surgeons used the cautery instead of the scalpel and Yperman in 1854 recommended scarifying the margins with a scalpel before suturing them with a triangular needle dipped in wax. The repair was reinforced by passing a long needle through the two sides of the lip and fixing the shaft of the needle with a figure-of-eight thread over the lip. Germanicus Mirault can be credited to be the originator of the triangular flap which was later modified by C.W. Tennison in 1952 and Peter Randall in 1959. In the late 50s, Ralph Millard gave us his legendary ‘cut as you go’ technique. The protruding premaxilla of a bilateral cleft lip too has seen many changes throughout the ages – from being discarded totally to being pushed back by wedge resection of vomer to finally being left to the orthodontists.

## THE AGE OF DENIAL AND IGNORANCE

Since in the ancient times man was ignorant of embryology and morphogenesis, his explanation for the existence of congenital deformities was based on a combination of religion, superstition, invention and charlatanism. The earliest traceable history of cleft lip and palate is that of horror and utter disbelief. In ancient times, many congenital deformities, including the cleft lip and palate, were considered to be evidence of the presence of an evil spirit in the affected child.[1] Facial deformities were most condemned and the infants were “removed from the tribe or cultural unit and left to die in the surrounding wilderness”, a practice that still prevails today in certain African tribes. In Sparta, the unfortunate newborns were abandoned on Mount Tagete, while in Rome they was drowned in the Tiber River or thrown off the Tarpeian rock. The noted philosopher Plato, far from opposing this practice, justified it in one of his dialogues in the *Republic*, explaining that it was a means of removing evil omens and preserving the soundness of the race. George Dorrance discussed the case of a mummy that had been reported in 1929 by Smith and Dawson in their work *Egyptian Mummies* published in London.[2] Thus Egyptians knew about the deformity. In the ancient Mediterranean civilizations these children were said to possess supernatural powers.[3]

Facial clefts were apparently unknown in Greece. Not a single reference of this deformity is found in the *Corpus Hippocraticus*, which represented a compendium of the medical knowledge of the period. This might lead one to suspect that this congenital deformity did not exist in the region. Tord Skoog, however, has demonstrated that such was not the case.[4] He describes a terracotta statuette found in 1969 in the Potters' Quarter of Corinth. Dating from 700–300 BC, the figurine portrays a clown with a complete cleft lip modelled in such meticulous detail that secondary defects of the premaxilla and the alae of the nose are clearly visible. Statuettes of figures with facial clefts can be seen at the Musée Guimet in Paris (Wagner Collection), the Museum für Folkerkunde in Munich, and in a group known as *Los Danzantes* at the archaeological site of Mount Alban in Mexico.

The learned archbishop of Uppsala in Sweden, Olaus Magnus[5] took the level of ignorance to its nadir when he in 1550 proclaimed that “however, there is one misfortune that many women meet with in pregnancy, either by eating or by leaping over the head of a hare; they bear children with a hare mouth, who have the lip permanently split between the mouth and nostrils, unless right from the beginning they sew a small piece of the breast of a very tender chicken, killed on the spot and still bleeding.” This state of ignorance is evident even up till 1889 when Keating[6] reported a series of congenital anomalies, including harelip, and opined that the anomalies were provoked in each case, by the mother looking a person with a similar deformity during her pregnancy.

## THE RETURN TO SANER SENSES

Fabricius ab Aquapendente (1537–1619) was the first to suggest the embryological basis of these clefts[7] when he suggested that in the development of the human foetus the upper lip only coalesces along the middle line at a very late stage. The most convincing explanation of the origin of the facial cleft in this period was furnished by Philippe Frederick Blandin (1838–96), who suggested that it resulted from a failure of the premaxilla and the maxillary segments to unite.[8–10] In 1808 Meckel[11] published his theory that the lips were formed from five separate processes which eventually united, three for the upper lip and two for the lower lip. William His of the University of Leipzig described that the embryological development of the mid-face resulted from the fusion of the five processes around the stomodeum, failure of any two of these parts to join would result in the formation of a different type of cleft, varying from unilateral and bilateral clefts to the rare cleft of the lower lip along the median line.[12,13] The first to note the congenital origin of the cleft was the thirteenth century physician Jean Yperman (1295– 1351). He classified the various forms of the condition and laid down the principles for their treatment.[14]

## RENAISSANCE ONWARDS

From the Renaissance to the 19^th^ century the knowledge and the surgery of cleft lip saw tremendous improvements. Pierre Franco, a pupil of Ambroise Paré never received a formal medical education but wrote two surgical texts based on his many years of experience, *Petit Traité* and *Traité des Hernies*.[15] The latter was published in 1561 and in it Franco discusses the cleft lip in ample detail devoting two chapters to the subject. He was the first to state the congenital nature of the malformation clearly, and referred to the unilateral harelip as the “lièvre fendu de nativité” (cleft lip present from birth). He provides a meticulous classification of various types of clefts, calling the bilateral harelip the “dent de lièvre” (hare's tooth) presumably because this condition was frequently accompanied by a marked protrusion of the premaxilla bone with its teeth.

## THE STORY OF LIP REPAIR

In 390 BC, there lived in China, a physician adept in the skill to correct this defect. An 18-year-old youth, Wey Young-Chi[16,17] who was born in the city of Jen in the province of Hupeh was the first patient. The surgery was performed in Nanking under the watchful eyes of Ying Chung-Khan, the Governor of the province and was a success. After his surgery Wey Young-Chi was recruited into the imperial army and quickly impressed General Lin-Yu, by helping to suppress a revolt. In due course of time, Wey himself rose to the rank of general and later became Governor of the Province of Yee. He eventually became Governor General of the six provinces. Throughout his life he affirmed that he would never have achieved so much if his cleft lip had not been repaired. This surely ranks as the very first of the string of success stories that constitutes the theme of the Smile Train project today!

According to Sterpellone and Salm El-Sheikh,[18] the Arab Albucasis and his fellow surgeons were reluctant to use the scalpel. They preferred to use hot metals (cautery) instead and recommended gold for haemostasis. They were however wise enough to realize that hot metal would cause more harm than good in the delicate tissue of a child's lip and practised a more gentle form of treatment. The cure recommended by Albucasis involved making a tiny incision into the lip, inserting a clove of garlic and leaving it for 15 hours. After removing the garlic, the margins of the defect were approximated with a bandage moistened with butter.

Yperman[14] called the deformity *sarte moude* (notched mouth) and recommended scarifying the margins with a scalpel before suturing them with a triangular needle dipped in wax. The repair was reinforced by passing a long needle through the two sides of the lip and fixing the shaft of the needle with a figure-of-eight thread over the lip. Heinrich von Pfolsprundt in 1460[19] made a significant contribution when in contrast to his predecessors, who only sutured the skin, he passed stitches through all the layers when repairing the cleft. He can thus be bestowed with the credit of first realizing the value of a good muscle repair.

In 1497 Hyeronimus Brunschwig, a military surgeon from Alsace after scarifying the margins of the cleft with scissors, applied a pinching clamp (*Zwickhafft*) or self-retaining clamp (*Telphaffen*), and then sutured the margins together with interrupted waxed stitches.[20] The sutures and clamp were left in place for some time, after which the wound was covered with a mixture made of egg and pulverized eggshell.

Pierre Franco[15] described the techniques of correction of both unilateral and bilateral cleft lips in *Traite des Hernies* very meticulously. He used dry sutures, pins and a triangular bandage. He emphasized that an accurate repair produced an unobtrusive scar, an outcome which was “particularly desirable when the patient was a girl”. Franco recommended that the cheeks be mobilized in the repair, but did not hesitate to resect the premaxilla. Ambroise Paré, Pierre Franco's teacher, was one of the greatest surgeons of the 16^th^ century; he conducted detailed studies on the anatomy of the lips and palate and introduced significant improvements in the technique of suturing. He is credited with the first illustration of an operation on a cleft lip and it appears in a work by Paré in *Les Oeuvres* showing the suturing method for cleft lip repair.[21]

Most surgeons till the early 19^th^ century were scarifying the margins of the cleft and suturing them together, employing various expedients to ensure good approximation of the edges. As can be imagined, the results were not always satisfactory. The vertical scar that formed invariably caused an ugly shortening of the lip. In 1844, Germanicus Mirault devised an ingenious method to circumvent this problem by introducing a triangular flap from the lateral side into a gap created by making a horizontal incision on the medial side.[22] This broke up the linear scar and introduced some extra tissue in an attempt to lengthen the lip. It also helped create a nostril floor. More than a century later Victor Veau stated “Mirault is the genius of cleft lip surgery”; and indeed his contribution was the most important since Franco's description of his two-step procedure.[23]

## TIMING OF THE REPAIR

This too was hotly debated as Hendrik van Roonhuysen of Amsterdam[24] and James Cooke of Warwick[25] felt that the operation should be carried out as soon as possible, when the patient was just three or four months of age as it is more dangerous to perform at an older age. However Leclerc[26] noted in 1701 that their “continual crying would hinder the reunion”. Both groups however agreed that the child should be kept awake before the operation so that he would fall asleep immediately afterwards, to help healing of the wound. The debate on the timing of surgery continued into the 19^th^ century. Andrea Ranzi introduced an important but hitherto neglected consideration.[27] He believed that while a simple harelip could be corrected shortly after birth, operations on more complex deformities should be postponed for up to five years. But he deserves credit for drawing attention to the psychological burden of the disfigurement as a crucial factor in the decision.

## REFINEMENT OF THE REPAIR

Gustav Simon reiterated the need for an accurate technique practised with the greatest delicacy and precision, the least amount of scar hypertrophy being sufficient to compromise the results.[28] Johan Fredrick Dieffenbach, who as a specialist in urethral operations had gained considerable experience in atraumatic techniques, was equally emphatic on the subject.[29] In the absence of safe and reliable general anaesthesia, surgeons were forced to acknowledge whatever results were obtained as satisfactory but all that changed soon after “Mirault's operation”[22] was widely adopted. Werner H. Hagerdon of Magdeburg who had studied under von Langenbeck, introduced a further improvement in 1848.[30] He recommended interrupting the vertical repair with a quadrangular rather than a triangular flap. This modification offered obvious advantages, particularly in the case of bilateral clefts, for it made the repair easier and by exerting pressure on the premaxilla helped to correct its protrusion. Hagerdon operated two babies within a week of their birth and was the first surgeon to do a single-stage bilateral cleft lip repair. A century later, in 1949, Hagerdon's technique was modified by Arthur Baker Le Mesurier,[31] then by C.W. Tennison in 1952[32] and Peter Randall in 1959.[33]

The modern-day lip surgeons who have left their indelible impression on the history of cleft lip surgery would surely include Victor Veau in the 1930s,[34] Tord Skoog who proposed a modification to Veau's approach and talked about boneless bone grafting of cleft alveolus,[35] Ralph Millard in the late 1950s for his monumental ‘cut as you go’ technique and several modifications and refinements,[36] Peter Randall in the 1960s for standardizing the triangular flap repair with accurate and reproducable measurements[33] and W.M. Manchester for his approach to the bilateral cleft in 1965.[37]

## THE BATTLE FOR THE PREMAXILLA

Tired of discarding the protruding premaxilla to facilitate the bilateral lip repair and paying a heavy price with a mid-face retrusion, surgeons were on the lookout for an alternative. In 1872, a radical method to correct this protrusion was developed by the Finnish surgeon Jacob August Estlander. He left the premaxilla intact and recommended a wedge resection of the vomer which allowed the protruding premaxilla to be pushed back.[38] Faltin, another Finnish surgeon, published his work in 1935 which recommended that the procedure be abandoned because it routinely led to serious maxillary retrusion.[39] Since Faltin published his work in Swedish, it took another 50 years for the predominantly English-speaking plastic surgery community to get his message through Millard's,[36] exhaustive compendium of 20^th^ century surgical procedures for clefts. Many a mid-face could have been prevented from going back had Faltin been conversant with the English language. Eventually the preoperative orthopaedic treatment devised by Ken McNeil and William Burston and adopted by many orthodontists replaced the vomerine resection, and this problem was resolved.[36]
